# Increased risk of acute kidney injury in the first part of an ultra‐trail—Implications for abandonment

**DOI:** 10.14814/phy2.15935

**Published:** 2024-04-29

**Authors:** Jean‐Charles Vauthier, Charlie Touze, Benoit Mauvieux, Corentin Hingrand, Pierre‐Louis Delaunay, Stéphane Besnard, Romain Jouffroy, Philippe Noirez, Patrice Maboudou, Cassandra Parent, Elsa Heyman, Mathias Poussel

**Affiliations:** ^1^ Département de Médecine Générale Faculté de Médecine Nancy France; ^2^ Département of General Practice Maison de Santé des Trois Monts Dommartin‐lès‐Remiremont France; ^3^ INTERPSY 5UR4432 Université de Lorraine Nnacy France; ^4^ UR 7480 VERTEX Université de Caen Caen France; ^5^ Service d'ORL Centre Hospitalier Universitaire de Caen Caen France; ^6^ Intensive Care Unit Ambroise Paré University Hospital, Assistance Publique – Hôpitaux de Paris, and Paris Saclay University Boulogne France; ^7^ IRMES – Institute for Research in Medicine and Epidemiology of Sport Institut National du Sport, de l'Expertise et de la Performance Paris France; ^8^ INSERM U‐1018, Centre de recherche en Epidémiologie et Santé des Populations Centre de recherche en Epidémiologie et Santé des Populations, Paris Saclay University Paris France; ^9^ Performance Santé Métrologie Société (EA7507) Université Reims Champagne Ardenne Reims France; ^10^ Univ. Lille, CHU Lille, Biologic et Pathologic Center Lille France; ^11^ ULR 7369 – URePSSS – Unité de Recherche pluridisciplinaire Sport Santé Société Univ. Lille, Univ. Littoral Lille France; ^12^ Institut de Recherches Cliniques de Montréal Montréal Québec Canada; ^13^ Institut Universitaire de France Paris France; ^14^ Department of Pulmonary Function Testing and Exercise Physiology University Hospital of Nancy, University Centre of Sports Medicine and Adapted Physical Activity, University of Lorraine Nancy France

**Keywords:** acute renal injury, biomarker, exercise physiology, extreme endurance, performance

## Abstract

Acute kidneys injuries (AKIs) have been described in marathon and trail running. The currently available data allows assessment of before/after comparisons but does not allow an analysis of what happens during the race. A multidisciplinary assessment protocol was performed during the first trail of *Clécy* (*Normandy France*) in November 2021. This allowed an initial assay to be carried out, then at the end of each of the 6 loops of 26 km, and finally after 24 h of recovery. The race extends over 156 km in hilly terrain and 6000 m of elevation gain (D+). The level of impairment according to the RIFLE classification was defined for each runner at each assay. Fifty‐five runners were at the start, and the per protocol analysis involved 36 runners (27 men and 9 women, 26 finishers). Fifteen (41.7%) of the riders presented at least one result corresponding to a “RIFLE risk” level. After 24 h of rest, only one runner still had a “RIFLE Risk”. The distance around the marathon seems to be the moment of greatest risk. For the first time, we find an association between this renal risk and the probability of abandonment. Many runners are vulnerable to kidney damage during long‐duration exercise, which is why it's important to limit risk situations, such as the use of potentially toxic drugs or hydration disorders. The consumption of NSAIDs (nonsteroidal anti‐inflammatory drugs) before or during an ultra‐distance race should therefore be prohibited. Attention should be paid to hydration disorders.

## 1 INTRODUCTION

The International Trail Running Association (ITRA) define a “trail race” like a “pedestrian competition open to everyone, which takes place in a natural environment with the minimum possible of paved roads (20% maximum)” (ITRA, 2023). The races are classified into six grades according to the number of kilometers effort. The term “ultra‐trail” is reserved for races over 80 km. Running is a popular sport. Fifty million Europeans and 60 million Americans run (Plard et al., 2021). In France, more than 3000 trail running events are organized each year.

But field experiences in real life are difficult to set up. To better understand the evolution of several physiological parameters during a race, a multidisciplinary protocol was proposed during a race, specifically dedicated to research (Mauvieux et al., 2022).

The practice of demanding sports such as ultra‐trail implies a relative conscious “risk‐taking” by the participants (exhaustion, traumatic injuries such as sprains, falls, blisters, mild digestive disorders, muscle cramps related to the effort…). However, participants are not aware of the possible risks for more critical dysfunction, such as renal failure, which is not acceptable and should be avoided. The physiological adaptation of the kidney to effort is a fundamental parameter to guarantee the safety of the runner. Acute renal failure can lead to reduced urination, digestive disorders, general fatigue, vomiting, and headaches. In severe forms, the course can be fatal. Cases of acute renal failure have been described in healthy ultra‐endurance athletes who have taken non‐steroidal anti‐inflammatory drugs (NSAIDs) (Chlíbková et al., 2015; Khodaee et al., 2015). NSAIDs can cause Acute Kidney Injuries (AKI) in healthy people or during acute events (such as acute gastroenteritis; Balestracci et al., 2015; Clive & Stoff, 1984; Misurac et al., 2013). The health authorities advise against the use of NSAIDs in patients at increased risk of renal failure (FDA, 2018; Ngo & Bajaj, 2023). It's not clear whether ultra‐trails can also lead to kidney risks in runners who don't take NSAIDs. Hoffman and Weiss (2016) suggested a prevalence of acute kidney injury (AKI) of 36% based on post‐race glomerular filtration rate calculated from serum creatinine levels (Poussel et al., 2020; Mingels et al., 2009), among 627 runners collected during 3 editions of the 161‐km Western States Endurance Run (Hoffman & Weiss, 2016). AKI was staged by the RIFLE classification (Risk, Injury, Failure, Loss of Kidney Function, and End‐stage Kidney Disease), a score usually employed in the intensive care unit to define the severity of AKI (Lopes & Jorge, 2013). While this RIFLE classification has to be determined from baseline values, the authors only based their evaluation on post‐race analyses. (Table 1). Conversely, by using baseline values in addition to post‐race values, Poussel et al. (2020) did not find any impairment of renal function in 54 runners on a 110‐km trail run in temperate climatic conditions. None of the 54 runners reached the first grade of the RIFLE classification considering the Cystatin C assay (another way for assessing GFR), and 1/54 reached the “risk” level using the serum creatinine assay. The two above‐mentioned discordant publications concerning renal risk induced by ultra‐trails were based on measurements performed only after the race and not during the race, making it difficult to understand renal physiology changes throughout such extreme exercise.

**TABLE 1:** RIFLE classification (simplified): Risk (named here RIFLEr), injury (RIFLEi), and failure (RIFLEf) of kidney function (Lopes & Jorge, 2013).

Class	GFR
RIFLEr = Risk	↑SCR × 1,5 or + ↓GFR > 25%
RIFLEi = Injury	↑SCR × 2 or + ↓GFR > 50%
RIFLEf = Failure	↑SCR × 3 or + ↓GFR > 75%

Abbreviations: GFR, glomerular filtration rate; SCr, serum creatinine.

Noteworthy, shorter distances, such as marathons, have been shown to trigger kidney damage. Mingels et al. (2009); McCullough et al. (2011); and Hewing et al. (2015) published studies on the marathon that supported a decline in kidney function after the race. The number of participants who reach the “risk” or “injury” stages in the RIFLE classification is between 30% and 50% (Lopes & Jorge, 2013). Mansour et al. (2017) also published on the kidney risk in marathons based on serum creatinine and on the urinary analysis of NGAL (Neutrophil Gelatinase‐Associated Lipocalin). Eighty‐two percent of marathon runners had acute renal failure, and 73% had tubular lesions. Finally, Atkins et al. studied the evolution of creatinine between the finish line and the following 24 h and found that renal stress persisted beyond 24 h, under the particular conditions of the Boston Marathon (high running speed and hot weather conditions; Atkins et al., 2022). Over a 60‐km trail, Lippi et al. (2012) found results comparable to those found in marathons with 38% prevalence of AKI (RIFLE at least “risk”) and a median decrease in GFR estimated by MDRD (Modification of Diet Renal Disease) at 31%.

Jouffroy et al. (2019) followed 47 subjects participating to an 80‐km trail, a distance close to the lower limit of an ultra‐trail. It was a longitudinal follow‐up (5 blood samples: baseline, 2 during the race, one after the finish line, and the last 9 days later). Interestingly, the top level of creatinine increased until the 53rd km and decreased at the end.

In view of the specific results of the studies on lower‐distance trails vs. the very little data available on ultra‐trails, there does not appear to be a dose‐damage relationship. Further studies including multiple measurements throughout ultra‐trail will be needed to understand whether very long distance is less dangerous or whether renal adaptation is actually disturbed at the start of the event (equivalent to the marathon distance) with subsequent new homeostasis setting in thereafter.

Thus, we aimed to find out whether ultra‐trail runners suffered from acute renal failure during their run. Repeated measurements during the race allow us to define periods of increased renal stress.

## 2 METHODS

The entire protocol including all the research has been published (Mauvieux et al., 2022). The launch of the study was authorized on October 26, 2021, under the trial number 21‐0166 after a favorable opinion from the *Comité de Protection des Personnes Ouest III* (21.09.61/SIRIPH 2G 21.01586.000009).

### 2.1 Subjects

Recruitment took place from January 2021 until September 2021 with an announcement on the social networks of the Scientific Trail of *Clécy*. Fifty‐five voluntary participants were selected, 43 (78%) men and 12 (22%) women, aged between 25 and 70 years old. After verification of the inclusion and exclusion criteria, they were invited to a videoconference outlining the entire protocol. The consent letter was provided for reading. On November 10, 2021, after a period of reflection and having been informed of all the details of the study, the participants signed the free and informed consent form. The runners were definitively included in the study after a medical check at the race site. Use of NSAIDs or renal‐disrupting drugs was exclusion criteria. A medical check was organized 24 h after the race and then 4–8 weeks later by teleconsultation.

### 2.2 The first trail Scientifique de Clécy 2021

The race started on November 11, 2021, at 2:30 p.m. It extended over 156 km in hilly terrain and 6000 m of elevation gain (D+). It was divided into six identical loops of 26 km and 1000 m D+. The runner had to be self‐sufficient in water and food between each refreshment point. A water supply was available halfway through the loop. At the end of each loop, runners had access to a refreshment station identical to that of a classic race (Beverages, food, and personal items). After this refueling, runners moved to the scientific zone (the stopwatch was paused for the duration of the scientific tests). After the tests, the runners started a new loop.

### 2.3 Blood samples

Blood samples were taken from the cubital fossa on the morning of the race, at the end of each of the 6 loops, at the end of the race, and 24 h after the race. Blood samples (2 mL) were taken in heparin tubes, centrifuged, and stored at −80°C for further analysis of plasma creatinine (enzymatic method including creatininase, creatinase, sarcosine‐oxidase, and peroxidase).

The Glomerular Filtration Rate (GFR) is calculated with the CKD‐EPI formula (without the ethnic criterion, unauthorized in France). Calculators are used to deduce the GFR from the assay, age, and gender (SFNDT, 2023). The serum creatinine assay can be disturbed by the muscle lysis that occurs in ultra‐trail (Arakawa et al., 2016; Kim et al., 2007; Shin et al., 2016). However, this marker remains the reference and is frequently used (Kashani et al., 2020).

### 2.4 Statistical analyses

For all values of GFR, a delta of the GFR at the end of the round (Delta T(n)/B) with respect to the baseline is calculated.

Relations between RIFLEr and age, gender, BMI, and quality of finisher (finisher vs. non‐finisher, the latter being defined as all causes of abandonment except for musculoskeletal injuries reasons) were sought.

For GFR measurements during the race per‐protocol (PP) analysis was conducted on all included participants who underwent a blood test before the race (Baseline). A modified intention‐to‐treat (mITT) analysis was conducted on all participants included, even those who did not have a baseline. For these, the value 24 h after the race served as a baseline.

Data was analyzed for each competitor to define which situations meet the RIFLE criteria. Continuous variables are presented as the mean and standard deviation. Outcome results were compared by using Fisher test for qualitative data. The analyses were performed using *BiostaTGV*, a threshold of *p* = 0.05 for two‐tailed tests being considered significant.

## 3 RESULTS

A total of 55 participants were included, 43 (78%) men and 12 (22%) women. The levels of competitors were diverse. The ITRA ranking ranged from 144 to 914.

The weather was cold and cloudy (Max. temperature 16°C and Min. temperature 7°C) (Nomades DC. Historique Météo, 2023).

**FIGURE 1:**
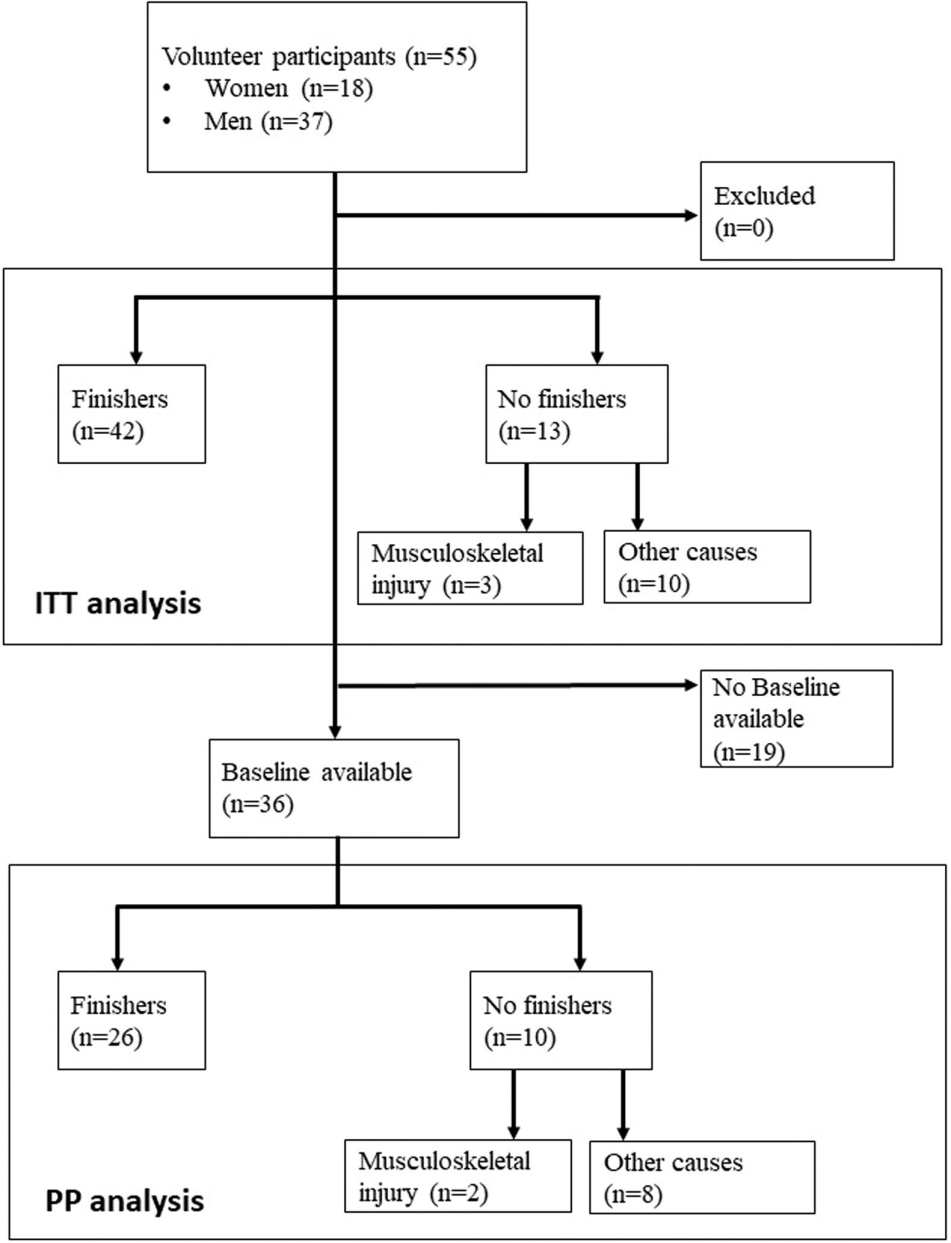
Flow chart.

The first man completed his course race in 17:11:02 (science time deducted). The first woman completed her race in 19:44:52 (science time deducted). The last runner completed his race in 35:55:21 (science time deducted; Figure 1).

### 3.1 The modified intention‐to‐treat analysis(mITT)

The modified intention‐to‐treat (mITT) analysis included those 55 subjects.

The average age was 43.60 (+/−9.65), and the average BMI was 21.97 (+/−2.13).

Thirteen (23.6%) dropped out, 3 for musculoskeletal injuries, 10 for other reasons (fatigue, hypothermia, gastric problems).

Eighteen (32.7%) competitors presented at least one GFR value corresponding to a RIFLE ‘risk’ classification. 37 (67.3%) runners had no value considered pathological (No RIFLE).

No one presented values corresponding to a more severe level of damage (injury or failure).

For one of his runners, the RIFLE analysis was not possible (no baseline before or after), but his GFR result at 54 mL/min/1.73m^2^ corresponds to a G3a stage of the EKD classification. It is counted among the RIFLE “risk”. (Table 2).

**TABLE 2:** Distribution of age, gender, BMI and finisher status among those with RIFLEr versus those without (in mITT).

MITT		Total	RIFLEr	No RIFLE
TOTAL (*n*)		55	18 (32.7%)	37 (67.3%)
Average AGE (years)		43,60 (±9,65)	45,06 (±10.05)	42,41 (±9.04)
Average BMI (kg/m^2^)		21,97 (±2,13)	22,35 (±1.73)	21,8 (±2.31)
Female (*n*)		12	3 (25%)	9 (75%)
Male (*n*)		43	15 (34.9%)	28 (65.1%)
No FINISHER	MSI (*n*)	3	0 (0%)	3 (100%)
	Other reasons (*n*)	10	6 (60%)	4 (40%)
	Total (*n*)	13	6 (46.2%)	7 (53.8%)
FINISHER		42	12 (28.6%)	30 (71.4%)

Abbreviations: MSI, Musculoskeletal injury; ns, no significant; OR, Other reasons.

No differences in demographic characteristics appeared according to RIFLE risk. However, the probability of RIFLEr tended to be higher among the athletes who dropped out for a reason other than musculoskeletal disorders compared with those who were finishers (*p* = 0.076).

The proportion of RIFLEr in each turn was counted against the number of measures available in that turn. The percentage of RIFLEr at each lap is presented in the following table and illustrated by the graphs which present this percentage and the average of SCrGFR according to the kilometers traveled.

**FIGURE 2:**
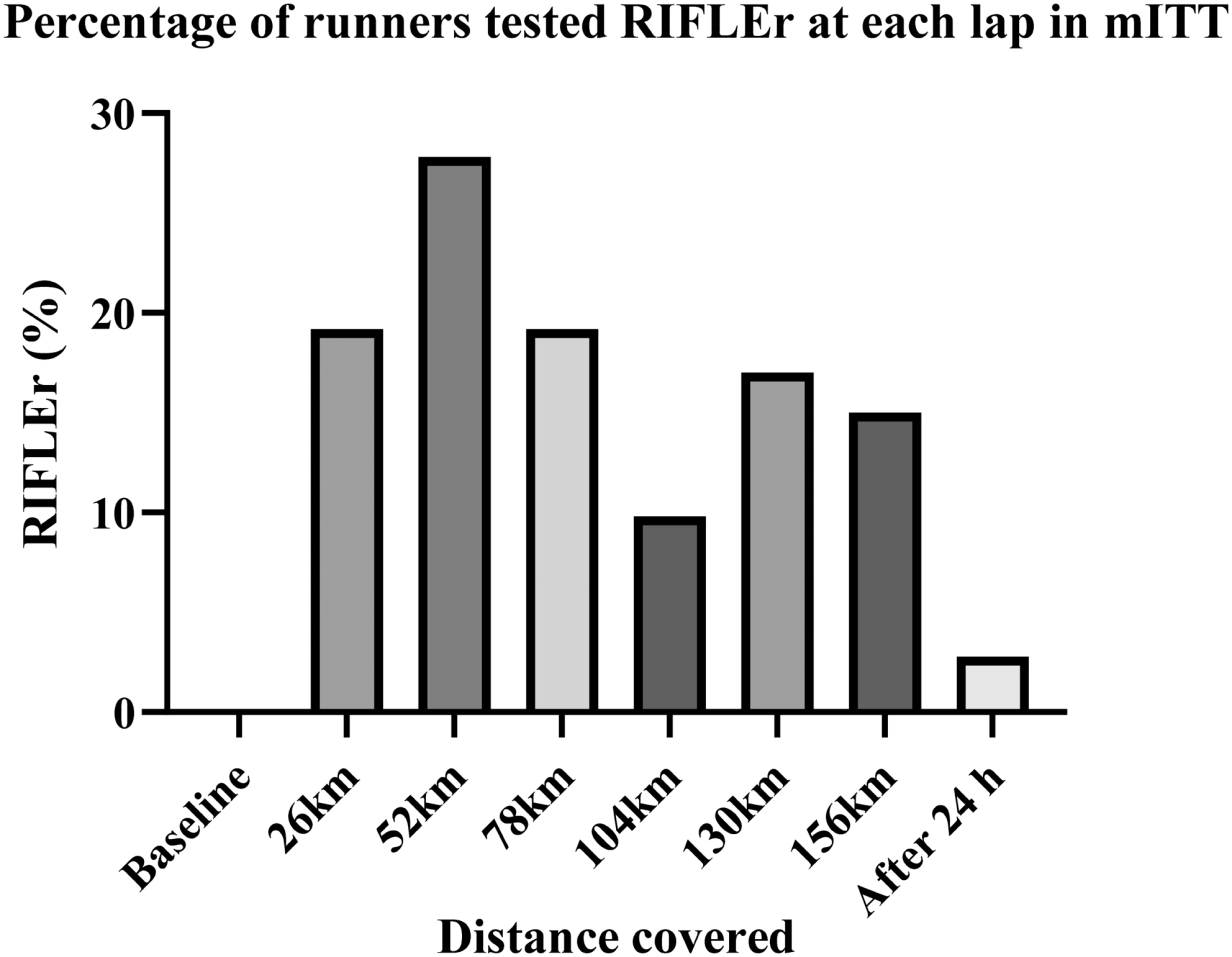
Percentage of runners tested RIFLEr at each lap in mITT.

**FIGURE 3:**
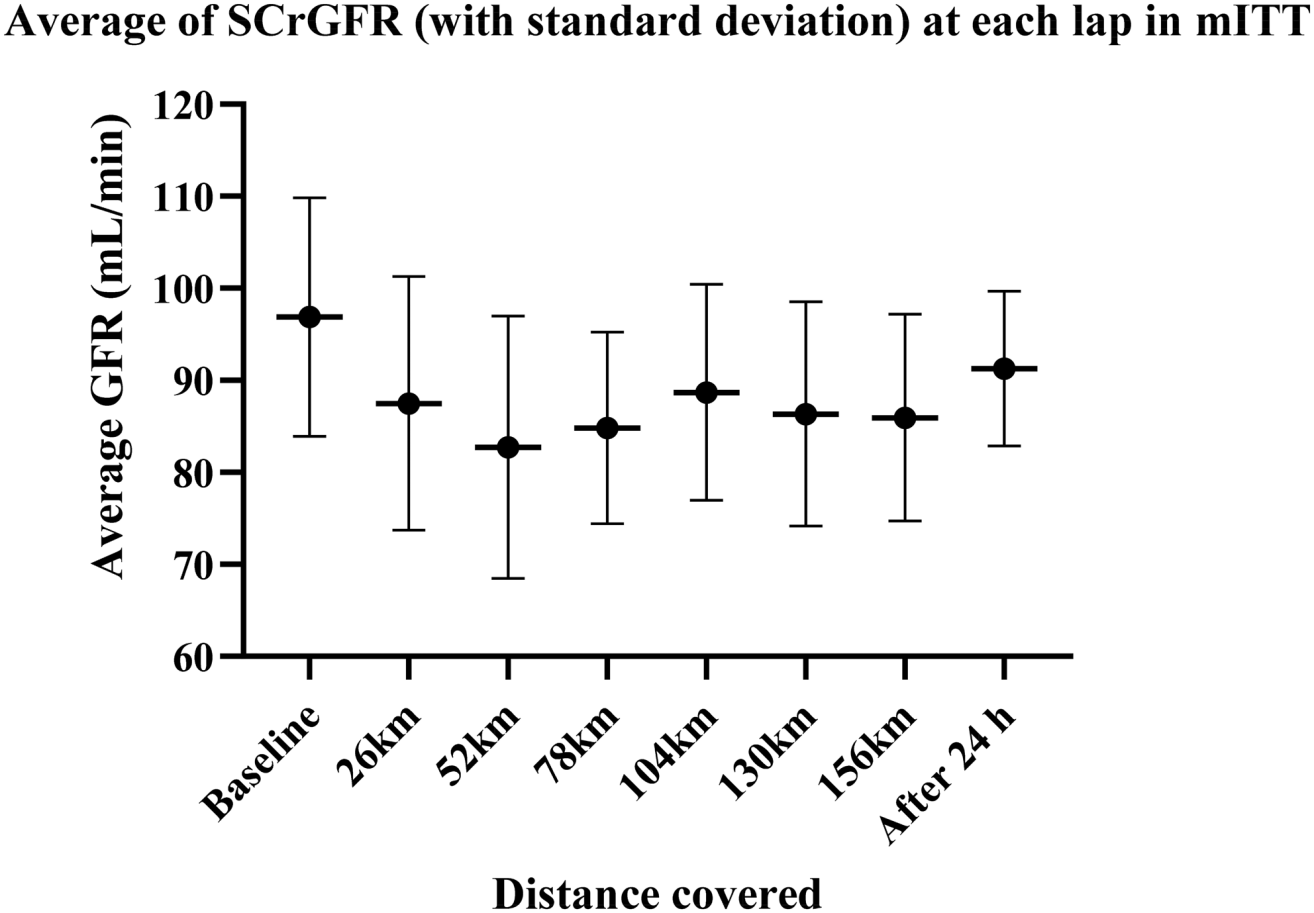
Average of SCrGFR (with standard deviation) at each lap in mITT.

Taking into consideration the timing of the first assay corresponding to a RIFLEr score, we found that the first moments at risk are all within the first 3 rounds. Eighty‐eight percent of the runners who presented values at risk during the race presented their first abnormal value in the first two laps. (Tables 3 and 4; Figures 2 and 3).

**TABLE 3:** Percentage of runners tested RIFLEr, Average of GFR (with standard deviation) at each lap in mITT.

mITT	nRIFLEr – stamples	RIFLEr (%)	Av GFR	Stan dev
Baseline		0	96,88	12,93
26 km	19–52	19,20%	87,49	13,78
52 km	16–54	29,60%	82,72	14,26
78 km	10–52	19,20%	84,81	10,41
104 km	5–51	9,80%	88,68	11,73
130 km	8–47	17,00%	86,34	12,15
156 km	6–40	15,00%	85,94	11,21
Rest 24 h	1–36	2,80%	91,26	8,4

Abbreviations: Av GFR: average of GFR.

**TABLE 4:** Timing of first RIFLEr assay.

First RIFLEr	Number mITT
Loop 1	10
Loop 2	6
Loop 3	2
Loop 4	0
Loop 5	0
Loop 6	0
Total	18

### 3.2 Per protocol analysis

Thirty‐six subjects met the conditions of the per‐protocol analysis.

The average age was 43.94 (+/−8.95), and the average BMI was 21.73 (+/−2.18).

Ten (27.8%) dropped out, 2 for musculoskeletal injuries and eight for other reasons (fatigue, hypothermia, gastric problems).

Fourteen (41.60%) presented at least one GFR value corresponding to a RIFLE ‘risk’ classification (RIFLEr).

No one presented with values corresponding to a more severe level of damage (injury or failure; Table 5).

**TABLE 5:** Distribution of age, gender, BMI, and finisher status among those with RIFLEr versus those without (per protocol).

Per protocol		Total	No RIFLE	RIFLEr
Total (*n*)		36	21 (58.3%)	15 (41.7%)
Average age (years)		43,94 (± 8,95)	42,24 (± 9.56)	46,33 (± 7.69)
Average BMI (kg/m^2^)		21,73 (± 2,18)	21,25 (± 2.32)	22,41 (± 2.18)
Female (*n*)		9	6 (66.7%)	3 (33.3%)
Male (*n*)		27	15 (55.6%)	12 (44.4%)
No FINISHER	MSI (n)	2	2 (100%)	0 (0%)
	Other reasons (n)	8	3 (37.5%)	5 (62.5%)
	Total (n)	10	5 (50.0%)	5 (50.0%)
FINISHER (*n*)		26	21 (80.8%)	5 (19.2%)

Abbreviations: MSI, Musculoskeletal injury; ns, no significant; OR, Other reasons.

The probability of being a RIFLEr if quitting (for a reason other than musculoskeletal disorders) was significantly higher from the probability of being a RIFLEr if being a finisher (*p*‐value 0.031).

The proportion of RIFLEr in each turn was counted against the number of measures available in that turn. The percentage of RIFLEr at each lap is presented in the following table and illustrated by the graphs that present this percentage and the average of SCrGFR according to the kilometers traveled.

**FIGURE 4:**
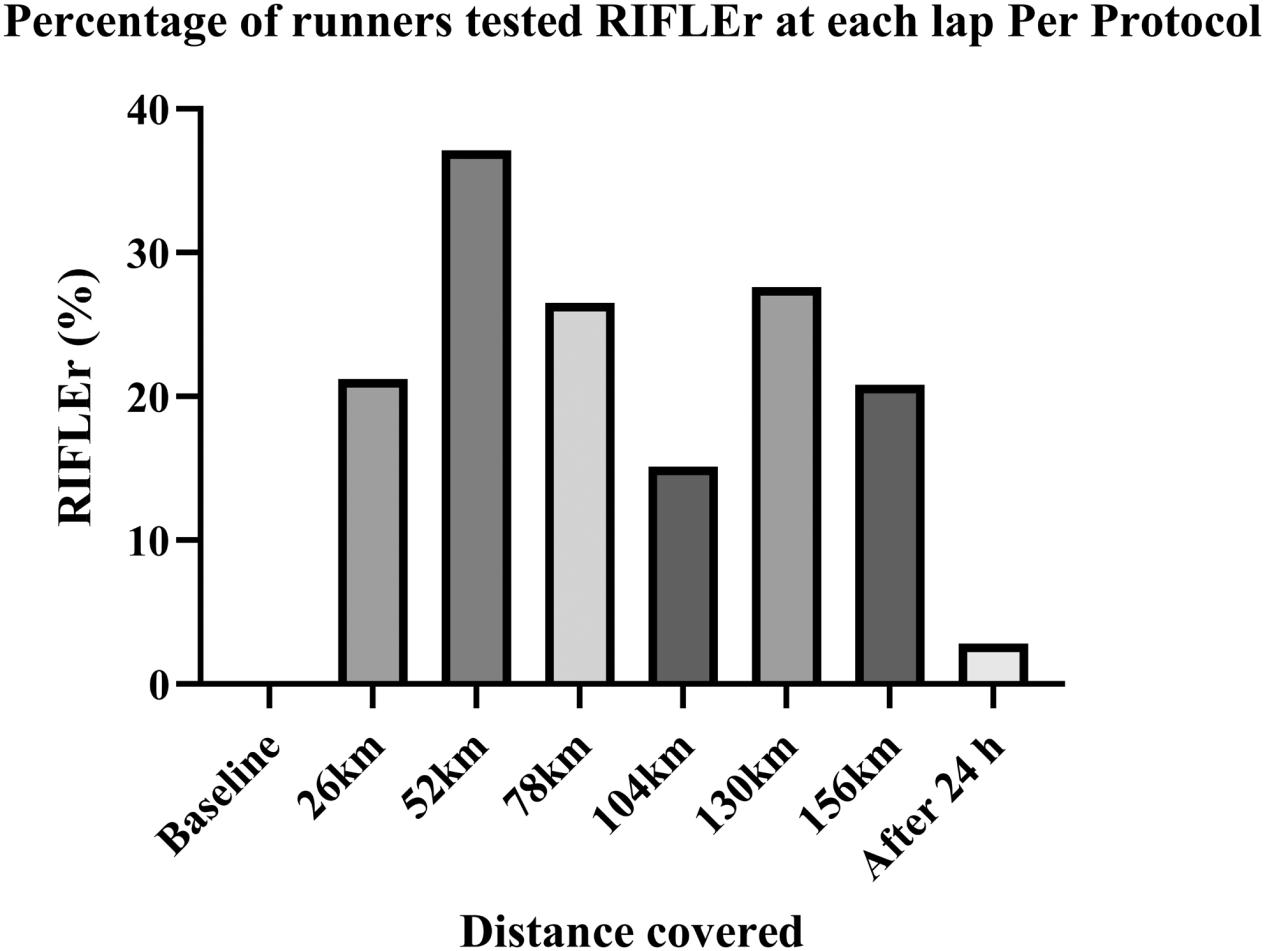
Percentage of runners tested RIFLEr at each lap per protocol.

**FIGURE 5:**
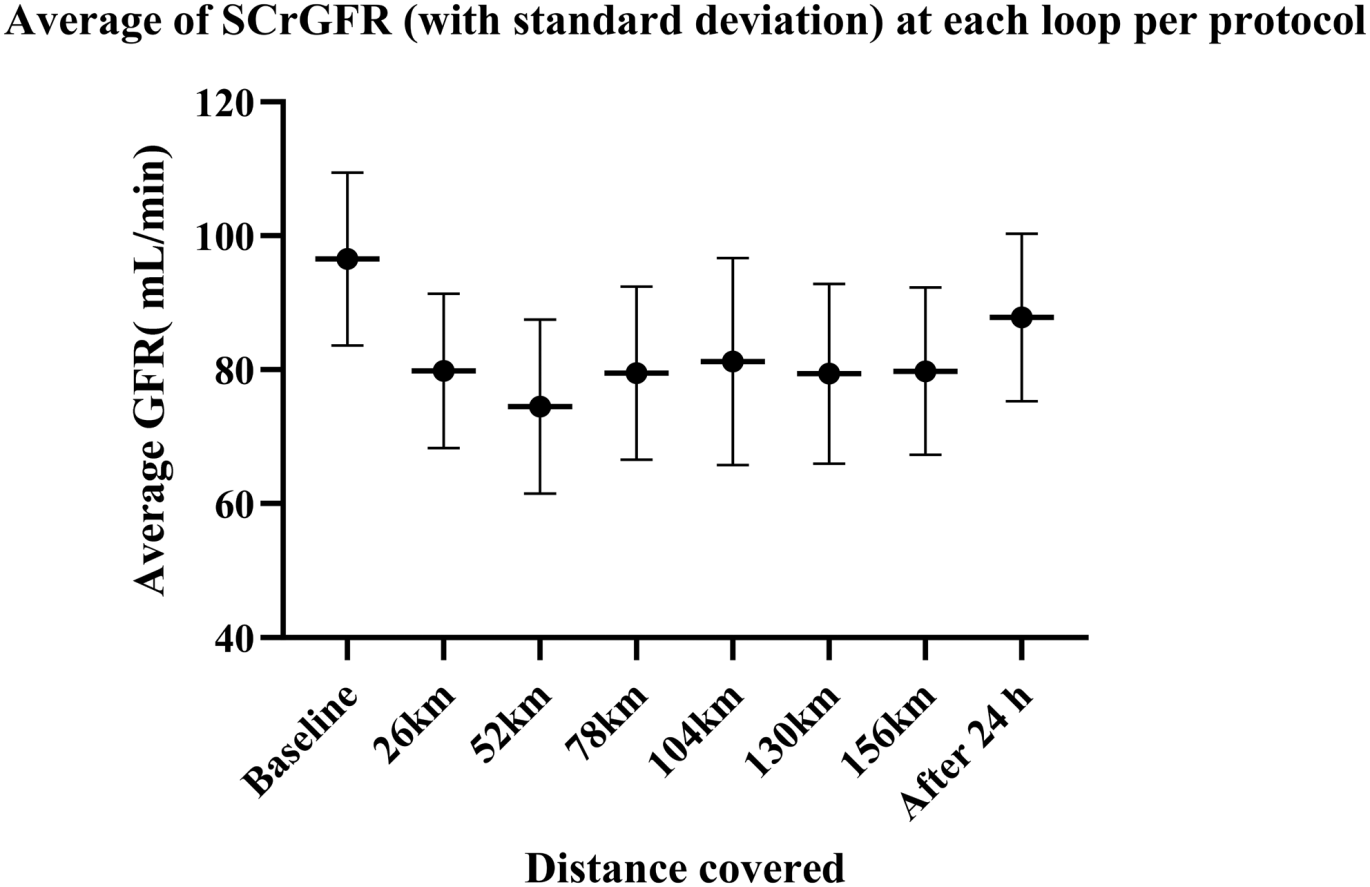
Average of SCrGFR (with standard deviation) at each lap per protocol.

Taking into consideration the timing of the first assay corresponding to a RIFLEr score, we found that the first moments at risk are all within the first 3 rounds. Ninety‐three percent of the runners who presented values at risk during the race presented their first abnormal value in the first two laps. (Tables 6 and 7; Figures 4 and 5).

**TABLE 6:** Percentage of runners tested RIFLEr, Average of GFR (with standard deviation) at each lap per protocol.

PP	nRIFLEr – stamples	RIFLEr (%)	Av GFR	Ecart type
Baseline		0	96,55	12,9
26 km	7–33	2120,00%	79,81	11,53
52 km	13–35	3710,00%	74,51	13,01
78 km	9–34	2650,00%	79,5	12,96
104 km	5–31	1510,00%	81,21	15,47
130 km	8–29	2760,00%	79,41	13,41
156 km	5–21	2080,00%	79,79	12,48
Rest 24 h	1–36	280,00%	87,8	12,52

Abbreviations: Av GFR: average of GFR.

**TABLE 7:** Timing of first RIFLEr assay.

First RIFLEr	Number PP
Loop 1	7
Loop 2	7
Loop 3	1
Loop 4	0
Loop 5	0
Loop 6	0
Total	12

## 4 DISCUSSION

In our population, which is comparable to the ultra‐trail population, the deterioration of renal function during the ultra‐trail effort (between 50% and 75% of the GFR of the GFR baseline) affected more than 40% of our sample, despite the cold and cloudy weather conditions during this fall weekend (l'actualité O fr: l'aventure au coeur de, 2019). Twenty‐four hours after the end of the race, most had recovered normal kidney function. Only one participant kept a RIFLEr score.

Disruption of renal function was associated with an increased risk of abandonment. This is new data. In fact, in the event of abandonment, it is often difficult to take a sample quickly after stopping. In the case of Poussel et al. (2020) study, runners who dropped out were far from the ephemeral laboratory. In our protocol, runners often chose to abandon at the end of their lap, close to the scientific base. Three stopped halfway through the lap, but a shuttle was ready to bring them back immediately. Until now, we thought that the kidney problems were asymptomatic and that the runners were not affected. We show here that these disturbances are associated with general reasons for abandonment (digestive disorders, fatigue, hypothermia) without, however, being able to establish causal links. The scope of our results remains fragile, with a significant per‐protocol analysis and a non‐significant intention‐to‐treat analysis. These results therefore provide information that needs to be verified in subsequent experiments.

The data available on marathon running seem to show a slightly greater risk than for ultra‐trailers. Our results confirm this with a higher RIFLEr rate around the marathon distance. It is also at this point in the race that the lowest level of average GFR is found (74.51 mL/min/1.73^2^ +/− 13.01). It is at the end of round 4 that the number of patients in a RIFLEr situation is the lowest and the average GFR is the highest. This is where the runners took the most rest. At the end of lap 5, the situation deteriorated again before improving towards the end of the race. It is therefore not a linear degradation that could be attributed only to muscle fatigue. Ninety‐three percent of RIFLEr cases are already declared after 2 laps (approximately equivalent to the marathon distance). In our series, if the runner has not yet been exposed to a deterioration in his renal function at mid‐race (here 3 loops), they have no risk of being exposed afterwards. The reasons for the peak of renal damage around 52 km remains to be investigated. It is possible that the kidneys are under intense stress at the start of the race, with faster running speeds and shorter break times. Behavioral adaptation related to fatigue leads the body to find, step by step, a new form of balance.

With a 40% risk of having transient functional renal failure during a trail run, the population of runners must be considered as a population at risk. Therefore, the consumption of NSAIDs should be strictly prohibited. In the same way, it is advisable to be vigilant with the major disorders of hydration (hypohydration or hyperhydration; Hew‐Butler et al., 2015). Unfortunately, the consumption of NSAIDs while trail running remains frequent, sometimes with serious consequences (Didier et al., 2017; Poussel et al., 1983). By inhibiting prostaglandins, NSAIDs block renal adaptations to exercise (Lucas et al., 2019). No runners took NSAIDs in our study.

And finally, the balance of kidney function was restored after 24 h of rest (except for one). It is not possible to determine whether this rapid recovery is a reassuring element that confirms that AKI (RIFLE “risk”) is functional and inherent to sports practice or whether exposure to situations of repeated renal risk results in subsequent damage. Poussel et al. (2020) demonstrated an increase in NGAL levels, which may indicate kidney injury, in 11.3% runners after a 110‐km trail run. Jouffroy et al. didn't confirm that on an 80‐km trail.

### 4.1 Study strength and limitation

The Multidisciplinary Protocol of the First Scientific Trail of *Clécy* 2021 is a unique experience. This design with six loops has made it possible to build an unprecedented pre‐, per‐ and post‐race database. The runners were experienced and were representative of the diversity of the packs of ultra‐trail runners (Age, Gender, BMI, ITRA Ranking). The per protocol analysis is carried out on 36 people. Up to eight blood samples could be taken. The main analysis uses the RIFLE classification, which makes it possible to use the baseline, as a control, limiting the biases associated with analytical performance.

“Real‐life” experimental conditions complicated the delivery and operation of vacutainers. Some samples were unusable. The organization in the scientific base did not make it possible to sample all of the competitors in each round. To make optimal use of all the results, two analyses (in mITT and PP) are proposed and find similar results. In this field study, we only had a small quantity of blood that could be analyzed, and we gave priority to measuring creatinine. We did not measure cystatin C, even though muscle damage could have influenced our creatinine values (Baxmann et al., 2008). Nevertheless, studies have shown that muscle damage worsens as the race progresses (Shin et al., 2016), suggesting that the drastic changes in creatinine levels observed in the first part of the ultra‐trail in our study were indeed induced by kidney damage. The raw results of SCr are published in mg/L without decimal. This impacts the precision of the results that are deduced and constitutes a bias.

In the design of the study, the runners were first allowed to refuel and then went to the laboratory to undergo a series of tests. The break devoted to the scientific tests was around 1 h, and blood samples were taken at the end of this break. The runners were able to rehydrate sufficiently, so we chose not to measure osmolarity in order to assess their state of hydration.

### 4.2 Conclusion and perspectives

For the first time, we show that the absence of kidney damage, as observed in some studies, masks a significant alteration in GFR occurring in the first part of the race and decreasing thereafter (Poussel et al., 2020). We confirm here that during their ultra‐trail effort, the runner has a high probability of finding themselves in a physiological situation of renal risk. This justifies immediately prohibiting the consumption of NSAIDs before or during a race. If the sports authorities do not pronounce this ban, then it is the responsibility of the doctors and pharmacists who accompany ultra‐trail runners to inform them of the danger that the consumption of NSAIDs entails. It is also necessary to ensure that the rules of water consumption “at thirst” are respected to avoid hydration disorders. The ultra‐trail is one of the rare sports where abandonment must be applauded. The probability of abandonment is increased in runners at renal risk. Stopping running is then the best choice, because stopping activity restores kidney function. Further research is needed on this subject. What happens when conditions are warmer? The lesional aspect of the renal damage by the analysis of the urinary NGAL could be an option in this way.

## AUTHOR CONTRIBUTIONS

J‐CV, CT, and MP contributed the conception and design of the study. BM, CH, P‐LD, SB, EH, and J‐CV contributed the conception and design of the complete protocol. RJ, PN, and J‐CV they took the blood, centrifuged and aliquoted the samples. CP, EH, and PM carried out the biological analyzes. J‐CV wrote the sections of the manuscript. All authors contributed to manuscript revision, read, and approved the submitted version.

## FUNDING INFORMATION

No funding information provided.

## CONFLICT OF INTEREST STATEMENT

The authors declare that the research was conducted in the absence of any commercial or financial relationships that could be construed as a potential conflict of interest.

## ETHICS STATEMENT

The study was authorized on October 26, 2021, under trial number 21‐0166 after a favorable opinion from the Comité de Protection des Personnes Ouest III (21.09.61/SIRIPH 2G 21.01586.000009).

## NEW AND NOTEWORTHY

No other study has succeeded in providing such extensive data “on racing”. The possibility of taking samples every 26 km under real race conditions is exceptional and requires considerable logistical effort. Moderate acute renal failure occurs in 41% of ultrarunners, particularly early in the race. This is why it is not advisable to take NSAIDs during an ultra‐trail. We identified a relationship between the onset of renal failure and the likelihood of abandoning the race.
